# Supplementary material for: Structural basis of VCP-VCPIP1-p47 ternary complex in Golgi maintenance
Source: Nat Commun. 2025 Aug 28;16:8025. doi: 10.1038/s41467-025-63161-3 (PMC12394548; doi:10.1038/s41467-025-63161-3)
Supplement: Supplementary file 1 — Supplementary Information [file 41467_2025_63161_MOESM1_ESM.pdf]

Supplementary Information

Structural basis of VCP-VCPIP1-p47 ternary complex in Golgi maintenance

Binita Shah[1,2], Moritz Hunkeler[1,2], Ariana Bratt[1,2], Hong Yue[1,2], Isabella Jaen Maisonet[1,2], Eric S. Fischer[1,2,*], Sara J. Buhrlage[1,2,*]

[*] Correspondence: Eric S. Fischer (E.S.F.), Sara J. Buhrlage (S.J.B.)

Eric_Fischer@dfci.harvard.edu

SaraJ_Buhrlage@dfci.harvard.edu

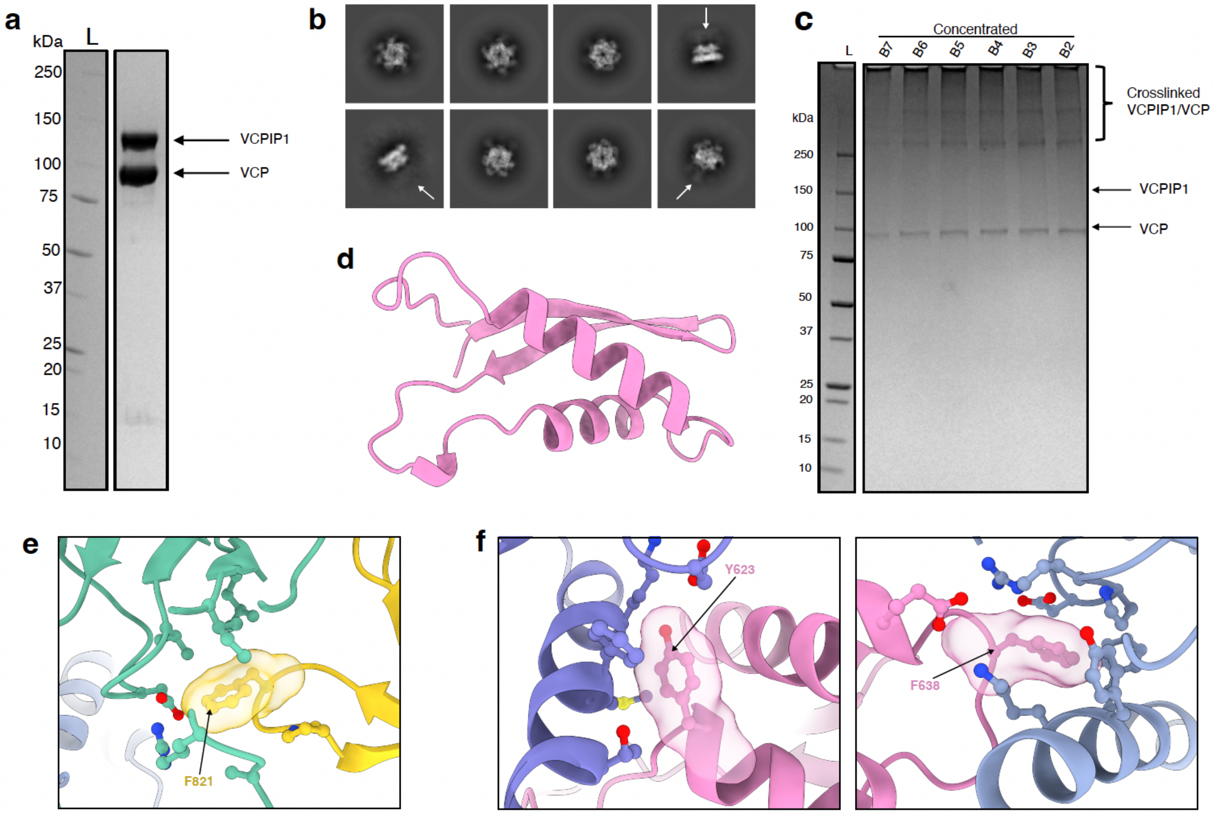

Supplementary Figure 1 | **Structural characterization of VCP-VCPIP1 complex. a** SDS-PAGE of purification of co-expressed VCP and VCPIP1 from Expi293 cells. **b** 2D classes from initial Talos Arctica session of the VCP-VCPIP1 complex without BS3 crosslinking. White arrows demonstrate VCPIP1 VCPID signal at the C-terminus of VCP. **c** SDS-PAGE of SEC fractions of BS3 crosslinked VCP-VCPIP1 complex that was concentrated for Titan Krios data collection. **d** Ribbon depiction of VCPIP1 VCPID. **e - f** Surface depiction of hydrophobicity found at the interaction sites for VCPIP1 UBX domain and VCPID, respectively. Hydrophobic areas are represented by surface mesh in yellow and pink, respectively.

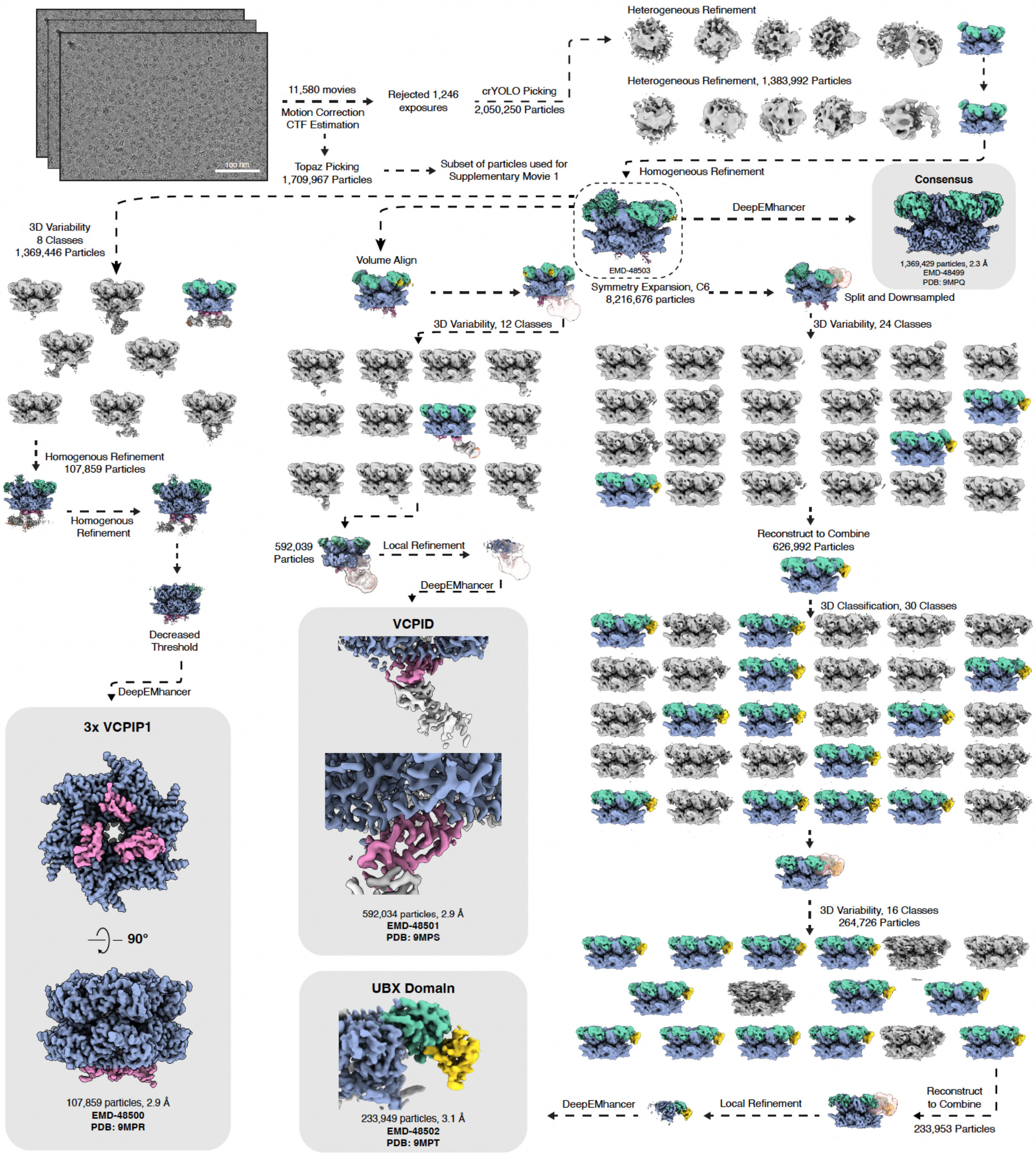

Supplementary Figure 2 | **Cryo-EM processing workflow for the VCP-VCPIP1 complex – Part 1.** Overview of processing workflow from raw micrographs to final maps. All steps were completed in cryoSPARC. All resolutions are given after post-processing. All colored volumes indicate selected classes of particles used for subsequent steps.

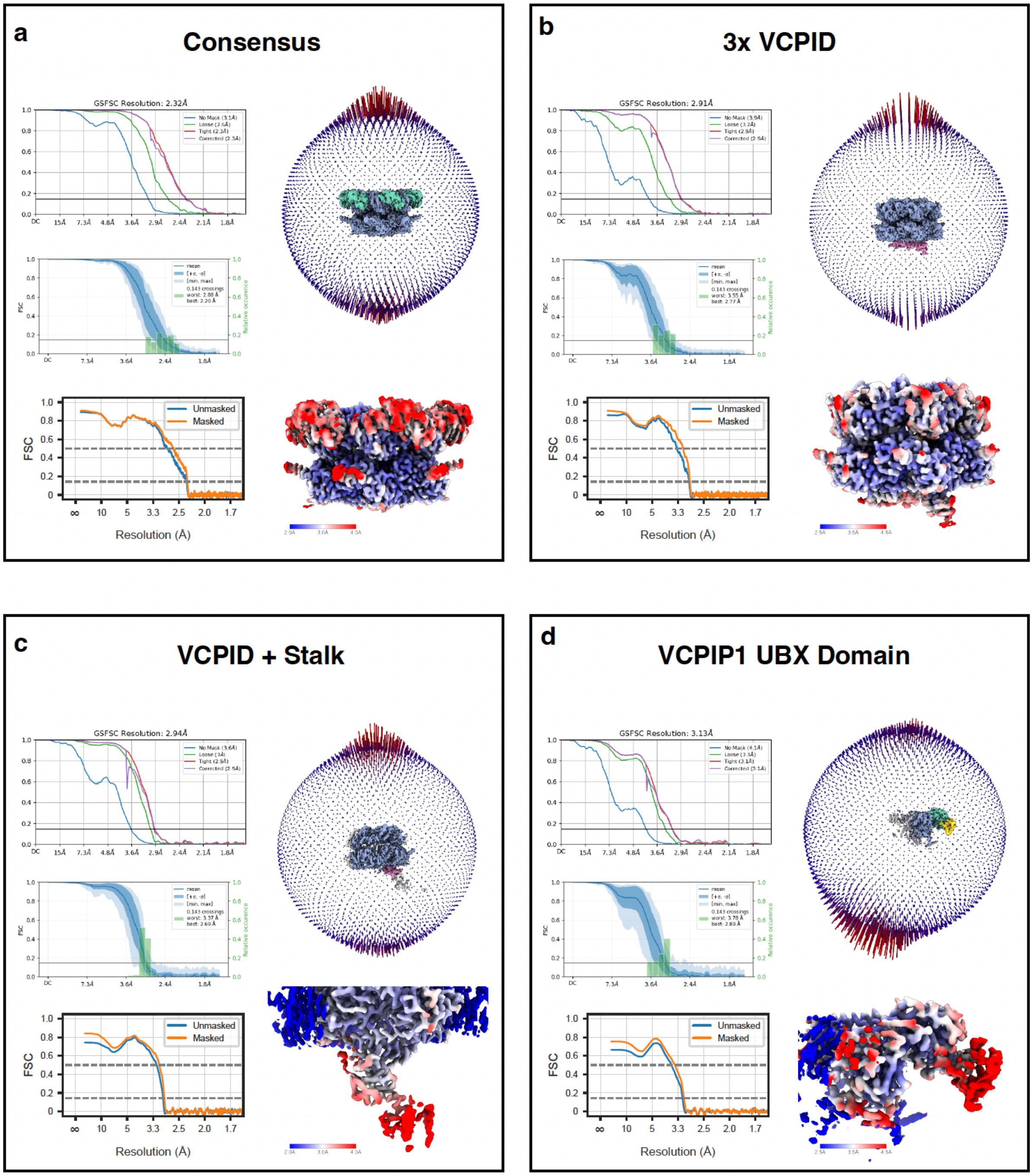

Supplementary Figure 3 | **Cryo-EM processing workflow for the VCP-VCPIP1 complex – Part 2. a – d** FSC plots (left top), 3DFSC plot (left middle), model-to-map FSC (left bottom), viewing direction distribution (right top) and main map colored according to local resolution (right bottom). (**a**) consensus EMD-48499 (**b**) 3xVCPID EMD-48500 (**c**) VCPID + stalk EMD-48501 (**d**) VCPIP1 UBX domain EMD-48502.

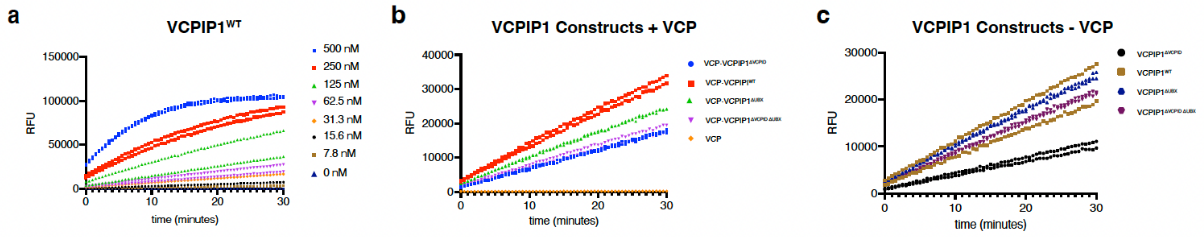

Supplementary Figure 4 | **DUB activity assay using ubiquitin rhodamine 110 (Ub-Rho) as substrate. a** Catalytic activity of VCPIP1^{WT} at different enzymatic concentrations and equal substrate (Ub-Rho) concentrations (500 nM). **b - c** Ub-Rho assay data reorganized with (**b**) and without (**c**) VCP at 62.5 nM enzyme concentration. (**b**) v_0 (VCP) = 3.81 RFU/min. All other values are depicted in Figure 2.

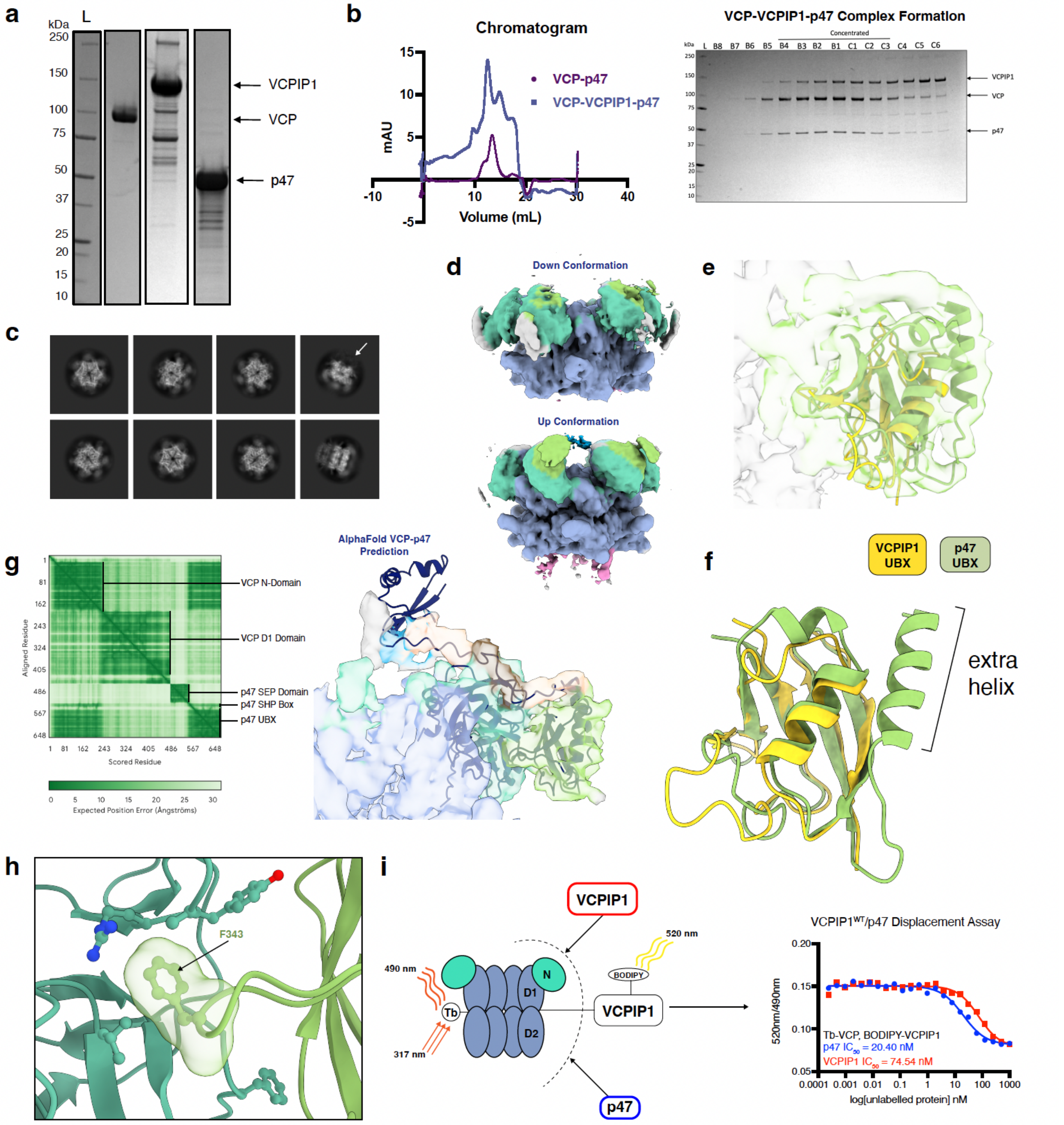

Supplementary Figure 5 | **Structural characterization of VCP-VCPIP1-p47 complex. a** SDS-PAGE gels depicting independently purified VCP (left), VCPIP1 (middle) and p47 (right). **b** SEC run on Superose6 Increase 10/300 GL (Cytiva) demonstrating traces of both VCP-p47 and VCP-VCPIP1-p47 complex formation. SDS-PAGE gel represents SEC fractions from VCP-VCPIP1-p47 complex formation, and the fractions concentrated for cryo-EM analysis on the Titan Krios. **c**

2D class averages of the VCP-VCPIP1-p47 complex from Titan Krios data set. **d** Classes from heterogeneous refinement job (highlighted in dashed green boxes in **Supplementary Fig. 6**) with N-domains of VCP in the down-conformation (top) and up-conformation (bottom). **e** VCPIP1 (yellow) and p47 UBX (lime green) domains individually fit in p47 UBX domain density (lime green, transparent). **f** VCPIP1 (yellow) and p47 UBX (lime green) domain alignment highlighting the extra helix on p47. **g** Highest rank prediction of p47 interaction with VCP N and D1 domains depicted by the resulting PAE plot (left) and model fit into density (right) **h** Surface depiction of hydrophobicity found at the interaction site for the p47 UBX domain and VCP N-domain represented similar to **Supplementary Fig. 1**. **i** Schematic and results of TR-FRET displacement assay of unlabeled VCPIP1 (red) and p47 (blue) displacing BODIPY-VCPIP1 from Tb-VCP.

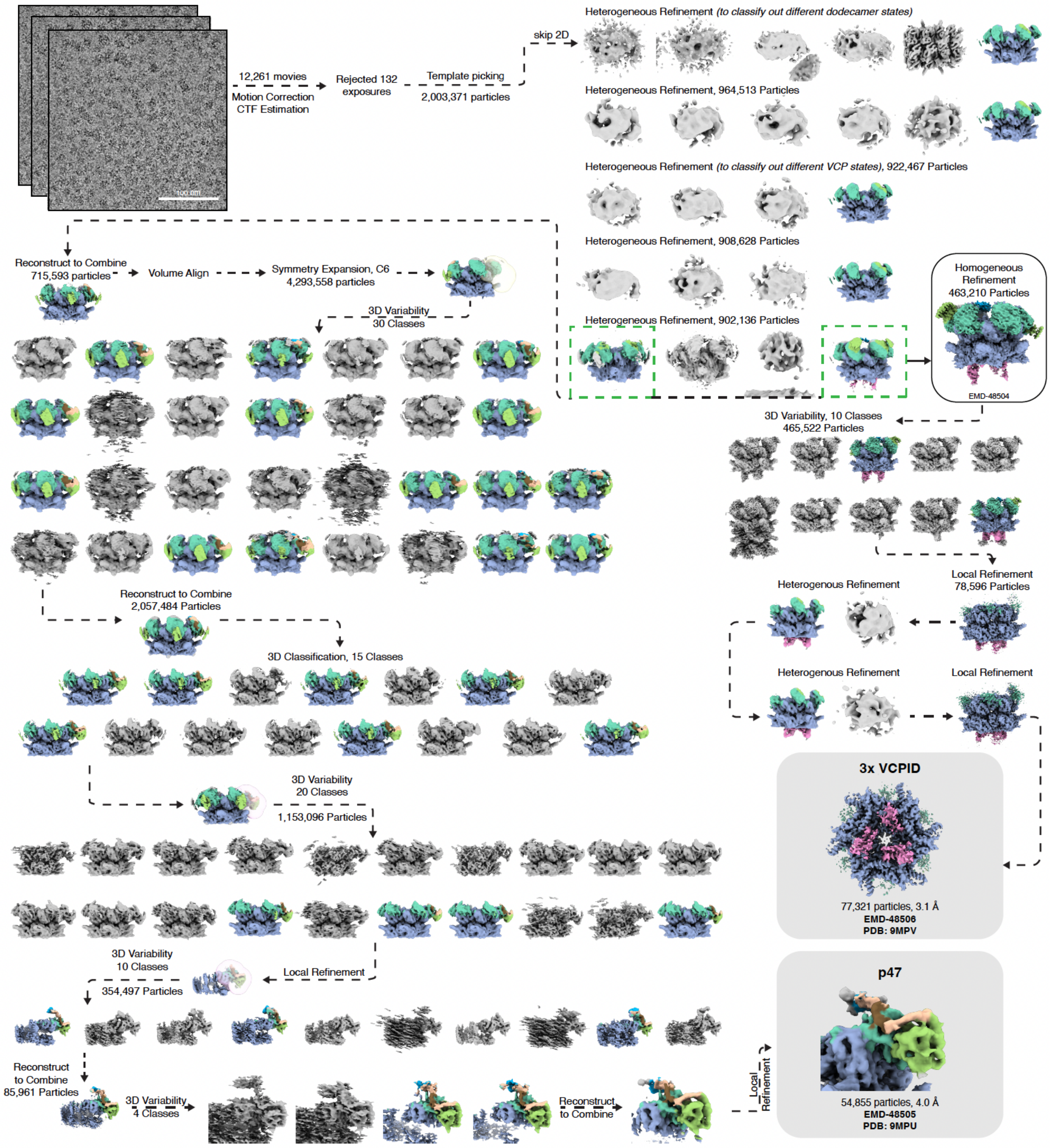

Supplementary Figure 6 | **Cryo-EM processing workflow for the VCP-VCPIP1-p47 complex – Part 1.** Overview of processing workflow from raw micrographs to final maps. All steps were completed in cryoSPARC. All resolutions are given after post-processing. All colored volumes indicate selected classes of particles used for subsequent steps.

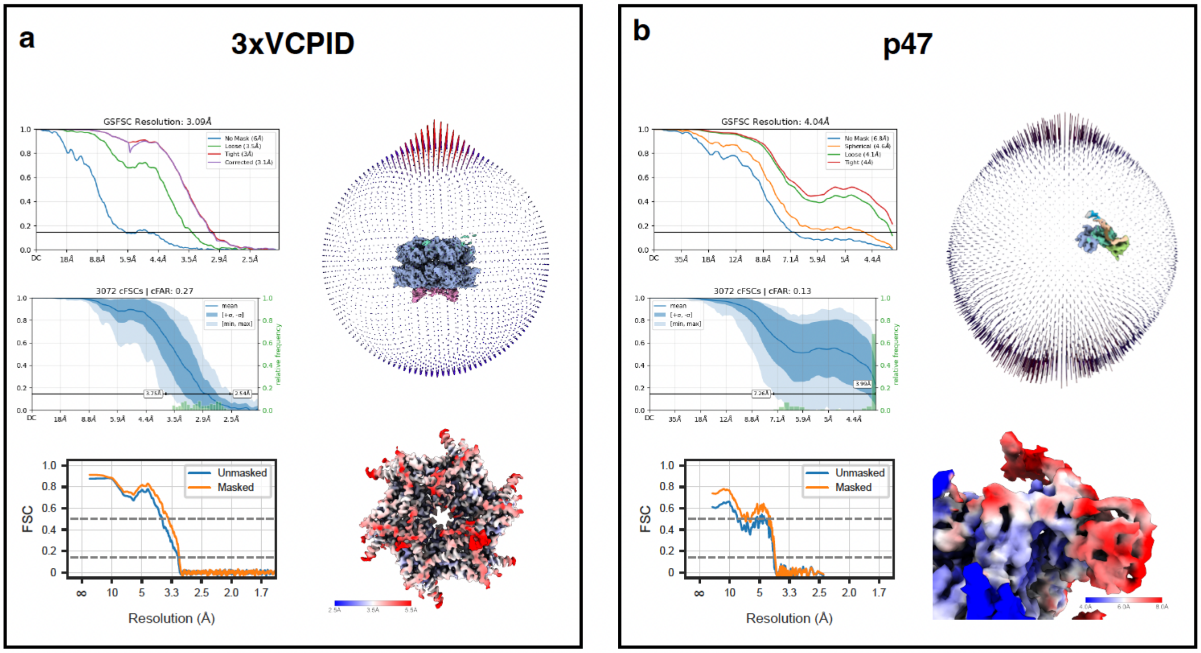

Supplementary Figure 7 | **Cryo-EM processing workflow for the VCP-VCPIP1-p47 complex – Part 2. a - b** FSC plots (left top), 3DFSC plot (left middle), model-to-map FSC (left bottom), viewing direction distribution (right top) and main map colored according to local resolution (right bottom). (**a**) 3xVCPID EMD-48506 (**b**) p47 EMD-48505.

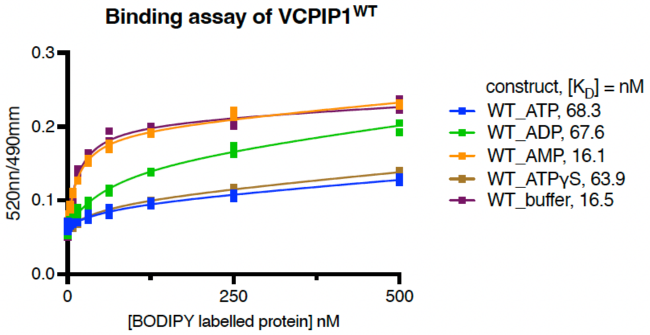

Supplementary Figure 8 | **Characterization of VCPIP1^{WT} binding to VCP in the presence of ATP analogs.** TR-FRET results of binding assay between Tb-VCP and BODIPY-VCPIP1 WT construct in the presence of no exogenous nucleotide, ATP, ADP, AMP and ATPγS.

Supplementary Fig. 1a

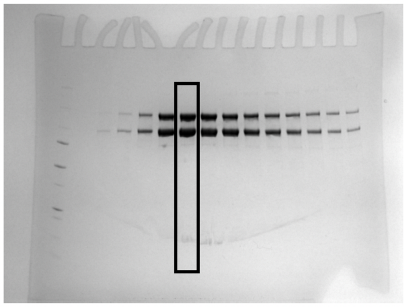

Supplementary Fig. 1c

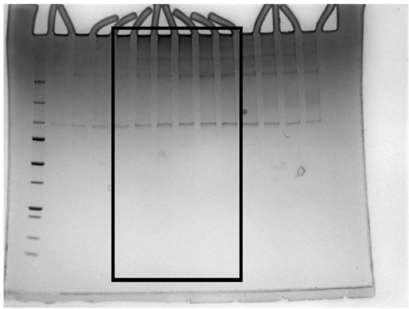

Supplementary Fig. 5a (left)

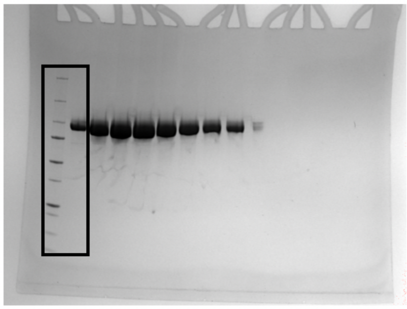

Supplementary Fig. 5a (middle)

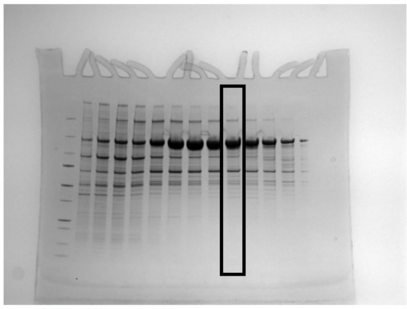

Supplementary Fig. 5a (right)

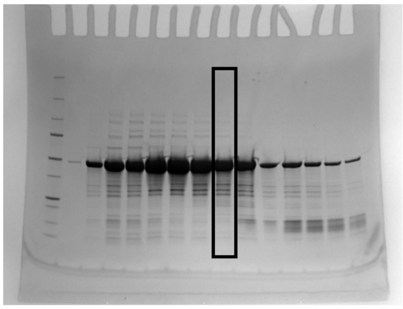

Supplementary Fig. 5b

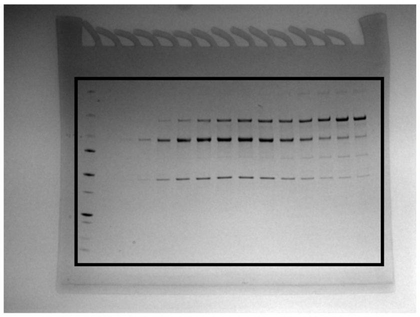

Main Figure 4a

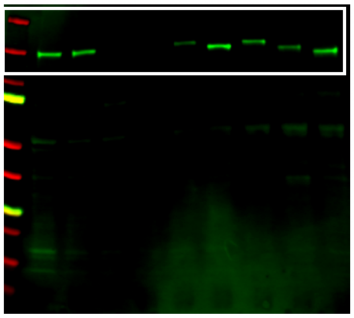

Supplementary Figure 9 | **Uncropped SDS-PAGE and western blots.** All original blots used in the supplementary figures with cropped regions marked (black box).

Supplementary Table 1 | **Cryo-EM data collection, refinement and validation statistics of VCP-VCPIP1 Complex**

**Data Set 1 – VCP-VCPIP1 Complex**					
Microscope	Titan Krios (Thermo Fischer Scientific)				
Voltage (kV)	300				
Camera	Gatan K3				
Magnification	105,000				
Pixel size (Å)	0.83				
Total electron exposure (e–/ Å^2)	53.69				
Number of frames (no.)	50				
Defocus range (µm)	-0.9 - -2.2				
Data Collection Software	SerialEM (v. 3.8.6)				
Energy filter slit width (eV)	20				
Micrographs collected (no.)	11,580				
Micrographs used (no.)	10,334				
Total extracted particles (no.)	2,050,250				
		Map 1	Map 2	Map 3	Map 4
		Consensus	3xVCPID	VCPID	UBX
EMDB accession code	EMD-48503	EMD-48499	EMD-48500	EMD-48501	EMD-48502
PDB accession code	-	9MPQ	9MPR	9MPS	9MPT
Final particles used (no.)	1,369,446	1,369,429	107,859	592,034	233,949
Symmetry	C1	C1	C1	C1	C1
Map resolution (Å, FSC 0.143)	2.9	2.3	2.9	2.9	3.1
Resolution range (Å)	2.5 – 6.4	2.0 - 5.5	1.8 - 7.9	2.5 – 6.5	2.7 – 6.4
Refinement Package	phenix.real_space_refine				
**Model composition**<br>**Non-hydrogen atoms (no.)**<br>**Protein residues (no.)**		 33,395<br>4,257	 27,895<br>3,563	 6,872<br>872	 4,117<br>523
Model-to-Map CC		0.84	0.85	0.81	0.81
Model-to-Map, FSC (Å, FSC 0.5)					
B factors (Å^2)<br>Protein (mean)<br>Water		 86.22<br>-	 86.29<br>-	 87.83<br>-	 97.96<br>-
**R.m.s. deviations**<br>**Bond lengths (Å)**<br>**Bond angles (°)**		 0.002<br>0.460	 0.003<br>0.476	 0.003<br>0.502	 0.003<br>0.510
**Validation**<br>**MolProbity score**<br>**Clashscore**<br>**Rotamer Outliers (%)**<br>**CaBLAM outliers (%)**		 1.61<br>6.78<br>1.49<br>2.21	 1.81<br>7.03<br>1.98<br>1.80	 1.93<br>8.60<br>1.91<br>2.88	 1.92<br>10.59<br>1.76<br>3.75
**Ramachandran plot (%)**<br>**Favored**<br>**Allowed**<br>**Disallowed**		 97.48<br>2.49<br>0.02	 96.80<br>3.17<br>0.03	 96.24<br>3.76<br>0.00	 96.89<br>2.91<br>0.19

Supplementary Table 2 | **Cryo-EM data collection, refinement and validation statistics of VCP-VCPIP1-p47 Complex**

**Data Set 2 – VCP-VCPIP1-p47 Complex**			
Microscope	Titan Krios (Thermo Fischer Scientific)		
Voltage (kV)	300		
Camera	Falcon4		
Magnification	165,000		
Pixel size (Å)	0.736		
Total electron exposure (e–/ Å^2)	49.22		
Number of frames (no.)	49		
Defocus range (μm)	-0.8 - -2.2		
Data Collection Software	EPU (v 3.7)		
Energy filter slit width (eV)	10		
Micrographs collected (no.)	12,261		
Micrographs used (no.)	12,129		
Total extracted particles (no.)	2,003,371		
		Map 1	Map 2
		3xVCPID	p47 UBX
EMDB accession code	EMD-48504	EMD-48506	EMD-48505
PDB accession code		9MPV	9MPU
Final particles used (no.)	463,210	77,321	54,855
Symmetry	C1	C1	C1
Map resolution (Å, FSC 0.143)	2.63	3.1	4.0
Resolution range (Å)	2.5 – 7.7	2.8 – 9.1	4.5 - 9.8
Refinement Package	phenix.real_space_refine		
**Model composition**<br>**Non-hydrogen atoms (no.)**<br>**Protein residues (no.)**		 28,483<br>3,641	 4,314<br>554
Model-to-Map CC		0.80	0.74
Model-to-Map, FSC (Å, FSC 0.5)			
B factors (Å^2)<br>Protein (mean)<br>Water		 90.93<br>-	 145.09
**R.m.s. deviations**<br>**Bond lengths (Å)**<br>**Bond angles (°)**		 0.002<br>0.456	 0.003<br>0.730
**Validation**<br>**MolProbity score**<br>**Clashscore**<br>**Rotamer Outliers (%)**<br>**CaBLAM outliers (%)**		 1.81<br>6.66<br>2.14<br>2.68	 1.83<br>11.85<br>0.00<br>4.09
**Ramachandran plot (%)**<br>**Favored**<br>**Allowed**<br>**Disallowed**		 96.83<br>3.17<br>0.00	 96.34<br>3.66<br>0.00
