# Supplementary material for: Structural basis of VCP-VCPIP1-p47 ternary complex in Golgi maintenance
Source: Nat Commun. 2025 Aug 28;16:8025. doi: 10.1038/s41467-025-63161-3 (PMC12394548; doi:10.1038/s41467-025-63161-3)
Supplement: Supplementary file 2 — Description of Additional Supplementary Files [file 41467_2025_63161_MOESM2_ESM.pdf]

**Description of Additional Supplementary File**

**File name:** Supplementary Movie 1

**Description:** Range of motion of VCPIP1 bound at the C-terminal of VCP. 3D variability analysis demonstrating that the stalk and OTU domains of VCPIP1 are highly flexible at the bottom of VCP. Movie created using ChimeraX.
